# Editorial: Current advances and challenges in understanding plant desiccation tolerance

**DOI:** 10.3389/fpls.2015.00768

**Published:** 2015-09-29

**Authors:** John P. Moore, Jill M. Farrant

**Affiliations:** ^1^Department of Viticulture and Oenology, Faculty of AgriSciences, Institute for Wine Biotechnology, Stellenbosch UniversityStellenbosch, South Africa; ^2^Department of Molecular and Cell Biology, University of Cape TownCape Town, South Africa

**Keywords:** resurrection plants, plant desiccation tolerance, seed desiccation tolerance, plant genome evolution, plant evolution

## Understanding plant desiccation tolerance – a global perspective

One of the most exciting and gratifying privileges of having edited this research topic on plant desiccation tolerance is that we received papers and reviews on resurrection plant species (particularly angiosperms) covering five continents, almost six, although unfortunately we did not quite get there. We were certainly incredibly fortunate for the kind responses of colleagues in Africa (Berjak and Pammenter, 2013, 2014), South America (Suguiyama et al., 2014), Asia (Mitra et al., 2013), Europe (Benina et al., 2013; Rakic et al., 2014), and Australia (Griffiths et al., 2014); for providing us with papers dealing with their own (favorite) particular resurrection plant species and the recent discoveries that they have made. It is with this in mind that we are moving toward a more global understanding of resurrection plants, and angiosperm species in particular, although often referred to as being particularly rich in diversity in southern Africa (Moore et al., 2009). We have noted that more and more studies are being made of resurrection species around the globe, as species are being uncovered in China such as *Boea hygrometrica* (this research topic, Mitra et al., 2013) and *Paraboea rufescens* (Huang et al., 2012) both in the Gesneriaceae and in South America Also in Brazil specifically, with *Barbacenia purpurea* (this research topic, Suguiyama et al., 2014) in the Velloziaeace. It is with this in mind that we are realizing more and more that plant desiccation tolerance in angiosperms is far less uncommon than previously suspected, and certainly has re-evolved as an adaptive feature on all continents (except for the South Pole, but this may well be provisional). We start off our quest for understanding plant desiccation tolerance with the green algae; here we are grateful for the first comprehensive review on this under-studied area, with a contribution from Holzinger and Karstens (2013). It is clear that algal cells were obviously the first “plants” to experience desiccation during land plant evolution. Far from following a simple single strategy, Holzinger and Karstens (2013) show that a variety of strategies appear to be employed to mitigate desiccation in both the Streptophyta and Chlorophyta lineages. We were hoping to include lichens and bryophytes (mosses), but these have been adequately covered in Moore et al. (2009). Our shift into the angiosperms, starts with an unlikely species, *Arabidopsis thaliana* (Djafi et al., 2013), however much is inferred, developed, tested using the Arabidopsis genetic model. In this case, an important area of angiosperm desiccation tolerance involves signaling (Moore et al., 2009), and Djafi et al. (2013) in their study focus on the phospoholipase C genes/proteins that are known to be triggered in response to dehydration. Djafi et al. (2013) have performed a thorough transcriptome study in *A. thaliana* using the presence of inhibitors that identified a set of *DREB2* (Dehyration Response Element Binding) regulatory genes involved in dehydration stress responses. Moving into resurrection plants we were fortunate to have received such a comprehensive review by Dinakar and Bartels (2013) of the various—omics studies (i.e., transcriptome, proteome, and metabolome) that has been performed on a variety of resurrection plants. Dinakar and Bartels (2013) have done an admirable effort to cover the variety of species evaluated at the molecular level; including *Craterostigma plantagineum, Haberlea rhodopensis, Xerophyta viscosa, B. hygrometrica, Sporobolus stapfianus*, and *Selaginella stapfianus*. A more specific review of the emerging model resurrection plant species *B. hygrometrica* from China was provided by Mitra et al. (2013). The authors conclude with a number of molecular factors that play a role in desiccation tolerance of *B. hygrometrica* before discussing future perspectives. We received two papers on resurrection plant species from the Balkan peninsula of Europe one on the genus Ramonda (Rakic et al., 2014) and the other on the species *H. rhodopensis* (Benina et al., 2013). The study by Rakic et al. (2014) focusses on the genus Ramonda and discusses aspects of their physiology, cytogenetics and biogeography. Benina et al. (2013) performed a comparative metabolic profiling study on *H. rhodopensis, Thellungiella halophyla*, and *A. thaliana* under cold stress showing that sugars, polyols, and organic acids accumulate as the main metabolites in the resurrection plant. Suguiyama et al. (2014) show that summer plants of *B. purpurea* are primed for desiccation, while winter plants show a two time-dependent response, involving metabolite accumulation, particularly the production of caffeoyl-quinic acids.

## The evolution of angiosperm resurrection plants – the secret in the seeds?

Farrant and Moore (2011) proposed that angiosperm resurrection plants acquired tolerance by re-activating their innate seed specific genetic elements in their vegetative tissues. Again we were fortunate to receive an excellent review on the lack of desiccation tolerance in recalcitrant (vs. orthodox) seeds by Berjak and Pammenter (2013). We were delighted to receive a novel study on the “seed desiccome” of *Medicago truncatula*, a seed model system, by Terrasson et al. (2013). This work identified 48 transcription factors involved in flowering transition, which the authors suggest were involved in co-opting the existing pathways during the evolution of desiccation tolerance in angiosperms. Given that programmed cell death is involved in seed physiology (Bewley et al., 2013) and processes such as senescence, it was therefore most satisfying to receive a review by Griffiths et al. (2014) on the role of senescence in resurrection plants. This topic has not been investigated in any depth, the authors draw on their own studies on *S. stapfianus*, to buttress their hypotheses on how senescence may be differentially activated and suppressed in resurrection plant evolution. The first sequenced genome of a resurrection plant, the Chinese *B. hygrometrica* (reviewed here by Mitra et al., 2013) was published this year by Xiao et al. (2015). This will pave the way for more functional genomic studies to elucidate the mechanisms underpinning desiccation tolerance in this as well as other species. It would seem we are only “*at the end of the beginning*” in our quest to understand the remarkable biological secrets of these fascinating plants.

### Conflict of interest statement

The authors declare that the research was conducted in the absence of any commercial or financial relationships that could be construed as a potential conflict of interest.
